# Coccidioidomycosis and Histoplasmosis in Immunocompetent Individuals: A Comprehensive Review of Clinical Features, Diagnosis, and Management

**DOI:** 10.7759/cureus.68375

**Published:** 2024-09-01

**Authors:** Harsh Babariya, Shilpa A Gaidhane, Sourya Acharya, Sunil Kumar

**Affiliations:** 1 Medicine, Jawaharlal Nehru Medical College, Datta Meghe Institute of Higher Education & Research, Wardha, IND

**Keywords:** management, diagnosis, fungal infections, immunocompetent, histoplasmosis, coccidioidomycosis

## Abstract

Coccidioidomycosis and histoplasmosis are endemic mycoses caused by the *Coccidioides* species and *Histoplasma capsulatum*, respectively. While these fungal infections are often associated with immunocompromised individuals, they pose significant risks to immunocompetent hosts. This review comprehensively analyzes these infections in immunocompetent individuals, focusing on clinical features, diagnostic approaches, and management strategies. The current understanding of coccidioidomycosis and histoplasmosis in immunocompetent individuals includes their clinical presentations, diagnostic methodologies, and treatment options. A literature review encompassed recent studies, clinical guidelines, and expert opinions. Data were analyzed to highlight critical aspects of the clinical manifestations, diagnostic processes, and management of these infections in immunocompetent patients. Coccidioidomycosis typically presents with pulmonary symptoms that may range from mild to severe and can include chronic and disseminated forms. Histoplasmosis also presents a spectrum of pulmonary symptoms with the potential for extrapulmonary dissemination. Diagnostic approaches for both infections involve clinical evaluation, serological tests, culture, and imaging studies. Management strategies include antifungal therapies such as fluconazole and itraconazole for coccidioidomycosis and itraconazole and amphotericin B for histoplasmosis, with treatment duration and monitoring tailored to the severity of the infection. Coccidioidomycosis and histoplasmosis can significantly affect immunocompetent individuals, with clinical presentations varying widely from mild to severe. Accurate diagnosis and appropriate management are crucial for optimal outcomes. This review underscores the importance of awareness and timely intervention in managing these endemic mycoses and highlights the need for continued research into better diagnostic and therapeutic approaches.

## Introduction and background

Fungal infections, or mycoses, encompass many diseases caused by fungi, ubiquitous in soil, air, water, and decaying organic matter [1]. Although most fungi are harmless to humans, certain species can invade tissues and cause infections, leading to various clinical manifestations. Mycoses can be categorized into superficial, cutaneous, subcutaneous, and systemic (deep) types, with systemic mycoses, also known as endemic mycoses, being particularly significant due to their potential to cause severe illness. Endemic mycoses are typically caused by fungi that thrive in specific geographic regions. They can cause systemic infections, particularly when their spores are inhaled and disseminated from the lungs to other body parts [2]. Among these endemic mycoses, coccidioidomycosis and histoplasmosis stand out as particularly important. These diseases are caused by dimorphic fungi, which exist in mold form in the environment and convert to a yeast form within host tissues [3]. Coccidioidomycosis, also known as "Valley fever," is caused by the *Coccidioides *species and is predominantly found in the arid regions of the Southwestern United States, Mexico, and parts of Central and South America. Histoplasmosis is caused by *Histoplasma capsulatum *and is endemic to the Ohio and Mississippi River valleys in the United States and parts of Central and South America, Africa, and Asia [4]. The study of these diseases is critical due to their geographic prevalence and potential to cause severe illness in both immunocompromised and immunocompetent individuals. While much of the existing research has focused on infections in immunocompromised populations, there is an increasing recognition of the significant impact these infections can have on immunocompetent individuals. A thorough understanding of their pathogenesis, clinical manifestations, diagnosis, and management in this population is essential for improving patient outcomes [5].

Coccidioidomycosis and histoplasmosis have traditionally been associated with immunocompromised states, where the body's natural defenses are weakened, making it easier for pathogens to establish infections [6]. However, these fungal infections are also relevant in immunocompetent individuals, who usually possess functioning immune systems but can still experience significant morbidity from these diseases. Several factors, including environmental exposure, occupation, and travel to endemic areas, influence the prevalence and incidence of these infections in immunocompetent populations [7]. For example, individuals who work outdoors in regions where these fungi are endemic, such as farmers, construction workers, and archaeologists, are at an increased risk of inhaling fungal spores, leading to infection. Even among healthy individuals, the clinical spectrum can range from mild, self-limiting respiratory illnesses to severe, life-threatening disseminated diseases [8]. From a public health perspective, these infections have broader implications beyond individual cases, impacting communities and healthcare systems. Coccidioidomycosis and histoplasmosis cases have risen in non-endemic regions, likely driven by climate change, migration, and travel [9]. This shift underscores the need for heightened awareness and improved diagnostic capabilities in areas where these diseases were previously rare. In addition, the economic burden of these infections can be significant, with costs associated with prolonged medical care, lost productivity, and, in severe cases, long-term disability. Understanding the relevance of these infections in immunocompetent individuals is crucial for developing effective public health strategies and ensuring prompt, appropriate medical interventions [10].

This review aims to comprehensively analyze coccidioidomycosis and histoplasmosis in immunocompetent individuals, focusing on the clinical features, diagnosis, and management of these infections. The primary objectives are to detail the spectrum of clinical presentations in immunocompetent individuals, ranging from asymptomatic infections to severe disease, and to examine the diagnostic approaches available, including laboratory tests, imaging studies, and differential diagnoses, with an emphasis on their application in immunocompetent hosts. Furthermore, this review will explore current management strategies, discussing the indications for antifungal therapy, treatment duration, and complication management. Recent advances in therapeutic options and ongoing research aimed at improving patient outcomes will also be highlighted. By synthesizing the current knowledge on these topics, this review will provide a valuable resource for clinicians, researchers, and public health professionals involved in the care and study of fungal infections, ultimately contributing to better prevention, diagnosis, and treatment strategies for coccidioidomycosis and histoplasmosis in immunocompetent individuals.

## Review

Coccidioidomycosis

Epidemiology

Coccidioidomycosis, commonly known as Valley fever, is primarily endemic to the southwestern United States and parts of Mexico. In the US, the disease is most prevalent in Arizona and California, where incidence rates are notably high. For example, in 2019, Arizona reported an incidence rate of 142.3 cases per 100,000 population, while California had a rate of 22.8 per 100,000 [11]. Other states, such as Nevada, New Mexico, Utah, and Washington, also report cases, although with lower incidence rates. In Mexico, particularly in the northern regions, states like Sonora, Baja California, Chihuahua, and Sinaloa are recognized as endemic areas. Studies using coccidioidin skin testing have shown high positivity rates in these regions, ranging from 13% to 74%, indicating a significant presence of the fungus in the environment [12]. Environmental and occupational risk factors are critical in the transmission of coccidioidomycosis. The fungus *Coccidioides*, which causes the disease, thrives in hot, dry, and dusty climates, making these areas particularly conducive to its spread. Activities that disturb contaminated soil, such as construction, agriculture, and archaeology, can release infectious spores into the air, increasing the risk of inhalation [13]. Dust storms and high winds in endemic regions further exacerbate this risk by dispersing fungal spores over wide areas. Certain occupations are at heightened risk due to exposure to these environmental factors. Construction, agriculture, and archaeology workers are particularly vulnerable, as their jobs often involve significant soil disturbance. In addition, individuals with weakened immune systems, such as those living with HIV/AIDS, organ transplant recipients, or those on immunosuppressive medications, are at greater risk of developing severe forms of the disease [13].

Pathogenesis

**Figure 1:**
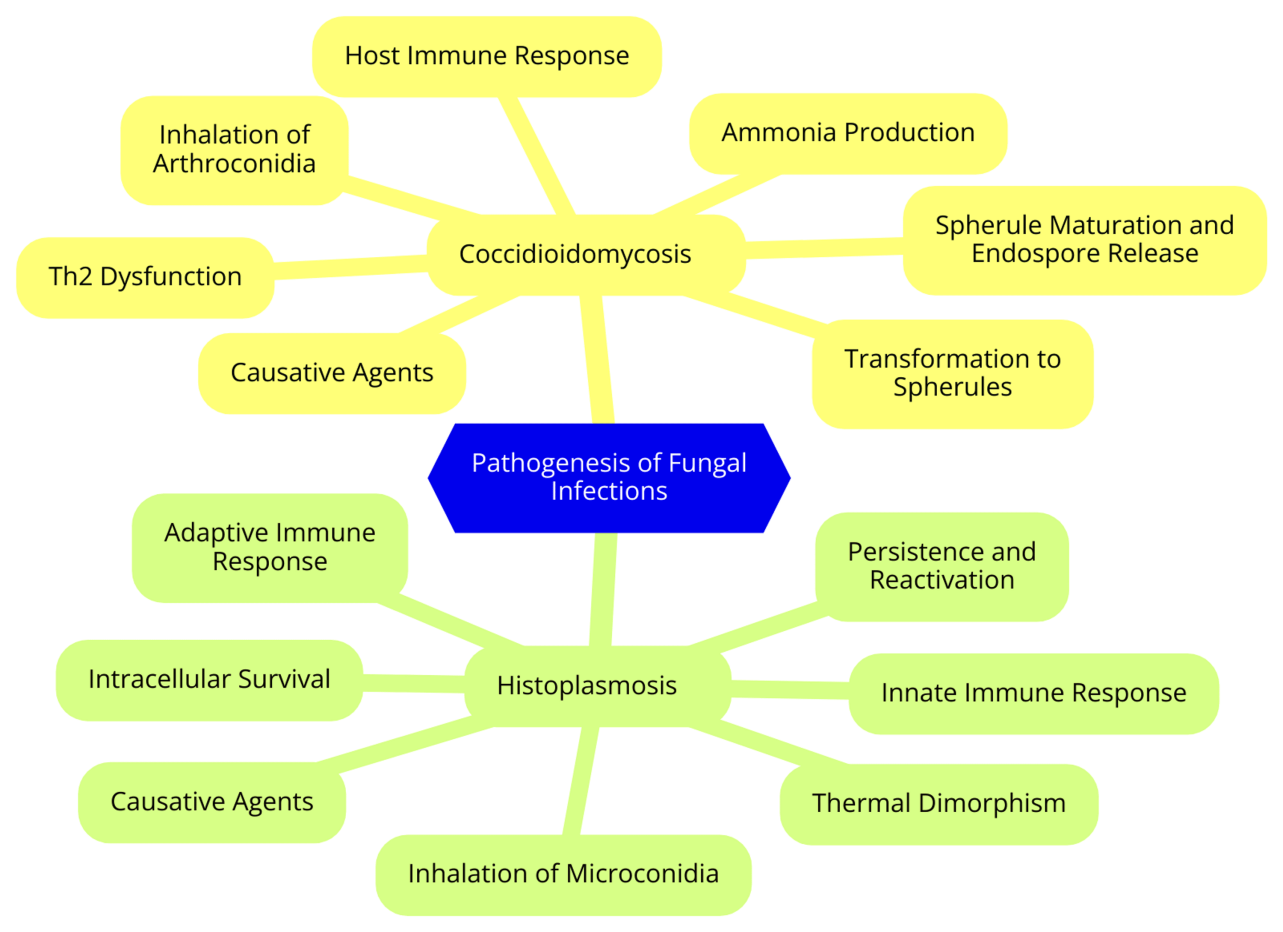
Pathogenesis of coccidioidomycosis, including the inhalation and dissemination of Coccidioides species Image credit: Dr. Harsh

Coccidioidomycosis is caused by two genetically distinct yet morphologically identical species of the dimorphic fungus *Coccidioides*: *Coccidioides immitis *and *Coccidioides posadasii*. *C. immitis *is primarily endemic to California's San Joaquin Valley, while *C. posadasii *is found in the southwestern United States, northern Mexico, and parts of Central and South America. Despite their genetic differences, both species exhibit similar pathogenicity, disease progression, diagnostic characteristics, and treatment responses [14]. The pathogenesis of coccidioidomycosis begins with the inhalation of infectious arthroconidia, the asexual spores of the fungus. These spores are typically released into the environment when soil is disturbed, becoming easily aerosolized. Once inhaled, the arthroconidia reach the lungs, where they transform into spherules, the parasitic form of the fungus. These spherules grow and mature within the pulmonary tissue, eventually rupturing to release endospores, which can further disseminate the infection. A key virulence factor of *Coccidioides *is its ability to produce ammonia, which alkalinizes the surrounding tissue, creating an environment conducive to fungal growth [15]. The host's immune response is crucial in controlling the infection. T-lymphocytes, particularly T-helper 2 (Th2) cells, are essential for mounting an effective defense against *Coccidioides*. In the early stages of infection, innate immunity, mediated by neutrophils, monocytes, and natural killer cells, helps to contain the arthroconidia that reach the lungs and manage the release of endospores. However, as the spherules increase in size, the effectiveness of these innate immune cells diminishes. This shift in immune response can lead to a more severe disease course, particularly in individuals with Th2 dysfunction or deficiency, who are at higher risk for extrapulmonary or disseminated disease [16]. The pathogenesis of coccidioidomycosis, including the inhalation and dissemination of the *Coccidioides *species, is illustrated in Figure 1.

Clinical Features

Coccidioidomycosis, primarily caused by *C. immitis *and *C. posadasii*, presents a spectrum of clinical features that can significantly impact the lungs and other organs. The disease is often marked by its pulmonary manifestations, particularly primary pulmonary coccidioidomycosis [17]. While most patients remain asymptomatic, symptoms typically appear one to three weeks after spore inhalation when they do occur. Common symptoms include fever, cough, chest pain, fatigue, chills, sputum production, and, in some cases, hemoptysis (coughing up blood). Physical examination may reveal scattered rales and dullness to percussion over affected lung areas. In addition, some patients may develop hypersensitivity reactions such as erythema nodosum, which is generally considered a favorable prognostic indicator [18]. Although many cases of primary pulmonary coccidioidomycosis resolve without complications, a subset of patients may progress to more severe forms. Complications can include the formation of nodular coin lesions in the lungs, which may be misdiagnosed as neoplasms or other infections. Cavitary lesions can also develop, posing a risk of rupture into the pleural space, potentially requiring surgical intervention. Recognizing and monitoring these complications are essential for effective management [19]. Extrapulmonary dissemination of coccidioidomycosis can occur, particularly in immunocompromised individuals or those with specific risk factors. Dissemination can affect various body systems, leading to skin lesions that may present as erythema nodosum or other rashes. Bone involvement, such as osteomyelitis, can result in localized pain and swelling. By contrast, central nervous system (CNS) involvement, particularly meningitis, represents a severe complication that can be fatal without prompt treatment. Symptoms of CNS involvement may include headache, fever, and neurological deficits, emphasizing the importance of early recognition and intervention [20]. The clinical presentation of coccidioidomycosis can be categorized into acute and chronic forms. Acute coccidioidomycosis typically presents with a rapid onset of respiratory symptoms and systemic signs, including flu-like symptoms such as fever, cough, and fatigue. Patients may also experience rheumatic symptoms, including arthralgias and myalgias. The presence of erythema nodosum, a skin manifestation associated with a robust immune response, is often seen in acute cases and is linked to a better prognosis [21]. By contrast, chronic coccidioidomycosis may develop in some patients, particularly those with underlying health conditions. This disease is characterized by persistent pulmonary symptoms, including a chronic cough, fatigue, and weight loss. Patients may also experience chronic cavitary pneumonia, involving the formation of lung cavities that may not resolve and can lead to further complications. Prolonged systemic symptoms, such as low-grade fever and malaise, can persist for months or even years following the initial infection, underscoring the need for ongoing monitoring and management [22].

Diagnosis

Diagnosing coccidioidomycosis requires a multifaceted approach that combines clinical evaluation, laboratory testing, imaging, and differential diagnosis. The diagnostic process begins with a high index of clinical suspicion, guided by a detailed patient history. Key historical factors include recent travel or residence in endemic areas such as the southwestern United States and exposure to occupational risks like construction or agricultural work involving soil disturbance. Clinicians should also assess the onset and duration of respiratory symptoms, fever, and systemic signs. A thorough physical examination may reveal respiratory distress, fever, and skin lesions, which could indicate disseminated disease [23]. Laboratory testing is essential for confirming the diagnosis of coccidioidomycosis. Serologic tests are commonly used to detect specific antibodies against Coccidioides. The presence of IgM and IgG antibodies can suggest recent or past infection; however, these tests can produce false-negative results, particularly in the early stages of the disease. The complement fixation test is valuable for assessing disease severity and monitoring treatment response. Fungal culture remains the gold standard for diagnosis, as isolating the *Coccidioides *species from clinical specimens, such as sputum, bronchoalveolar lavage, or tissue, provides definitive proof of infection. However, cultures may take weeks to yield results. Molecular diagnostics, particularly polymerase chain reaction (PCR), offer a rapid and sensitive detection of *Coccidioides *DNA from various specimens, including blood and tissue samples, making PCR especially useful in cases of disseminated disease [24]. Imaging studies play a crucial role in evaluating pulmonary involvement. A chest X-ray is often the first imaging modality used. It may reveal pulmonary infiltrates, nodules, or cavitary lesions, although these findings are nonspecific and can resemble other pulmonary conditions. A chest CT scan is recommended for a more detailed assessment, as it provides superior visualization of lung structures and can help identify complications such as abscesses, cavitations, or lymphadenopathy. This advanced imaging is particularly useful when disseminated disease is suspected [25]. Finally, considering differential diagnoses is critical, as coccidioidomycosis can mimic other conditions. Bacterial pneumonia is a common differential due to its similar presentation with cough, fever, and chest pain. Tuberculosis, especially in endemic regions, must also be ruled out, particularly with a history of exposure. Other fungal infections, such as histoplasmosis, can present with comparable respiratory symptoms. In addition, sarcoidosis, lung cancer, and other fungal infections (e.g., blastomycosis or cryptococcosis) should be considered, depending on the patient's geographical and clinical context [26].

Management

Managing coccidioidomycosis involves a comprehensive approach that includes antifungal therapy, vigilant monitoring, and addressing potential complications. The choice of antifungal medication, such as fluconazole or itraconazole, depends on the severity of the disease and the patient's clinical condition. Fluconazole is commonly used as the first-line treatment for moderate to severe pulmonary coccidioidomycosis, disseminated cases, and chronic pulmonary infections, particularly when patients exhibit symptoms. Itraconazole is an alternative, especially in cases where fluconazole is ineffective or not tolerated, and is also effective for chronic pulmonary infections requiring long-term management [27]. The duration of antifungal therapy varies based on the infection's severity. Treatment typically lasts three to six months for mild to moderate cases but may be extended depending on clinical improvement and radiological findings. In severe cases, initial treatment may involve intravenous amphotericin B, followed by a transition to oral fluconazole or itraconazole, with therapy often lasting six to 12 months or longer. Chronic or disseminated cases may require long-term therapy, sometimes extending for a year or more. Regular monitoring during treatment is essential, including assessments of clinical symptoms; liver function tests, particularly with itraconazole due to its potential hepatotoxicity; and serum drug levels to ensure therapeutic concentrations [28]. Complications such as meningitis and chronic pulmonary coccidioidomycosis require specialized management. Coccidioidal meningitis is a serious complication that demands aggressive treatment, typically involving long-term fluconazole therapy, often for life, to prevent recurrence. Patients with chronic pulmonary coccidioidomycosis may also require prolonged antifungal therapy, with treatment lasting up to a year or longer, depending on symptom persistence. In some cases, surgical intervention may be necessary for patients with significant pulmonary complications, such as abscesses or cavitary lesions [29]. In addition to meningitis and chronic pulmonary infections, disseminated disease presents unique challenges. Patients with disseminated coccidioidomycosis may require a combination of antifungal therapy and surgical intervention, particularly if the infection has spread to multiple sites. Asymptomatic pulmonary nodules may not require treatment, but symptomatic nodules should be evaluated for possible antifungal therapy or surgical resection. Managing coccidioidomycosis is multifaceted and necessitates a tailored approach to ensure effective treatment and optimal patient outcomes [30].

Prognosis and Outcomes

Coccidioidomycosis in immunocompetent individuals presents distinct prognostic factors and outcomes. Age is a significant prognostic factor, with studies indicating that mortality rates increase with advancing age. For example, one cohort study found that the mean age of immunocompetent patients was around 73 years, which correlated with a higher mortality rate [31]. The initial severity of the disease at diagnosis also critically influences outcomes. Patients who require hospitalization or admission to an intensive care unit (ICU) have higher mortality rates. A study reported that 39% of immunocompetent patients died within six months of diagnosis, with inpatient status being a significant risk factor [31]. The presence of comorbidities, such as diabetes mellitus and chronic lung disease, can negatively impact prognosis. Chronic conditions, in particular, may complicate recovery and increase the likelihood of severe disease progression. In addition, the overall immune status of the patient, even among those considered immunocompetent, can influence outcomes. Genetic predispositions or prior health conditions may affect the body's ability to respond to the infection effectively [32]. Long-term follow-up is essential for monitoring potential complications or recurrences. Regular assessments help identify late-onset symptoms or chronic pulmonary issues that may develop after initial recovery. The recurrence of coccidioidomycosis in immunocompetent individuals is relatively low, with studies indicating a recurrence rate of about 5%, primarily occurring in patients with some form of immunocompromise at the time of recurrence. This suggests that while recurrence in immunocompetent patients is possible, it is more likely in those with underlying health issues [33]. Even after recovery, some patients may experience long-term respiratory issues that can impact their quality of life. Monitoring for chronic pulmonary symptoms post-infection is recommended. In summary, while many immunocompetent individuals recover from coccidioidomycosis, age, initial disease severity, and comorbid conditions significantly influence prognosis. Long-term follow-up is crucial, and although recurrence is uncommon, it tends to occur more frequently in those with compromised immune systems [33].

Histoplasmosis

Epidemiology

Histoplasmosis is a globally distributed disease with endemic regions on every continent, although it is primarily associated with the Americas [34]. The disease is most prevalent in North America in the Midwestern and Southeastern United States, particularly in the Ohio and Mississippi valleys. It is most commonly found in Latin America in Venezuela, Ecuador, Brazil, Paraguay, Uruguay, and Argentina [35]. Within Brazil, endemic areas are located mainly in the Midwestern and Southeastern regions, where prevalence rates range from 4.4% to 63.1% and 3.0% to 93.2%, respectively. These high-endemicity regions generally share environmental conditions characterized by a moderate climate and constant humidity [36]. Histoplasmosis is primarily contracted through the inhalation of microconidia from the fungus *Histoplasma capsulatum*. Risk factors for infection include environmental exposures that disturb soil contaminated with bird or bat droppings, such as those found in chicken coops, construction sites, or caves [37]. Occupational exposure also plays a significant role, with half of all histoplasmosis outbreaks reported in occupational settings. Between 1938 and 2013, more than 100 outbreaks, involving approximately 3,000 cases, were reported across 26 US states and Puerto Rico. Birds, bats, or their droppings were identified in 77% of these outbreak settings [38]. In endemic regions, pinpointing the exact source of exposure can be challenging, as symptoms may result from the reactivation of a latent infection. Over half of the global population is estimated to reside in regions endemic to *H. capsulatum *[39].

Pathogenesis

Histoplasmosis is caused by the fungus *H. capsulatum*, which has two primary varieties: *H. capsulatum *var. *capsulatum *and *H. capsulatum *var. *duboisii*. *H. capsulatum *var. *capsulatum *is the more common variety, particularly in the Americas, where it thrives in environments rich in organic nitrogen, such as soil contaminated with bird or bat droppings [40]. When inhaled, the microconidia of *H. capsulatum *var. *capsulatum *transforms into a yeast form at body temperature (37°C), a critical adaptation that enhances its pathogenicity. By contrast, *H. capsulatum *var. *duboisii *is less prevalent and primarily associated with African histoplasmosis, often leading to more severe disease presentations, especially in immunocompromised individuals [41]. The infection begins when *H. capsulatum *microconidia or mycelial fragments are inhaled into the lungs. Once inside, these spores convert to the yeast form, enabling them to survive and replicate within host macrophages. This transformation, triggered by the temperature shift from the external environment to the human body, is essential for the organism's virulence. The fungus' ability to persist and multiply within the hostile environment of the immune system is a key factor in its pathogenicity [41]. The host immune response is crucial in controlling histoplasmosis. Initially, the innate immune system responds by recruiting neutrophils and macrophages to the infection site. Although macrophages engulf the yeast cells, *H. capsulatum *has evolved mechanisms to evade destruction, allowing it to proliferate intracellularly. The adaptive immune response, particularly the activation of CD4+ T lymphocytes, is vital for the effective clearance of the infection. These T cells activate macrophages through cytokines such as interleukin-12 (IL-12) and interferon-gamma (IFN-γ), enhancing their fungicidal capabilities [42]. However, *H. capsulatum *can persist in a dormant state within macrophages, posing a risk of reactivation in immunocompromised individuals. The fungus' ability to manipulate the host's immune response further complicates the infection, allowing it to survive and replicate despite the host's defenses. This complex interaction between the pathogen and the host immune system highlights the challenges in managing histoplasmosis, particularly in vulnerable populations. Understanding the pathogenesis of *H. capsulatum*, including the differences between its varieties and the host immune response, is essential for developing effective diagnostic and therapeutic strategies [41].

Clinical Features

Histoplasmosis, caused by the fungus *H. capsulatum*, manifests in a broad spectrum of clinical presentations, ranging from mild pulmonary symptoms to severe disseminated disease. The most common form is acute pulmonary histoplasmosis, which typically resembles a flu-like illness, presenting with symptoms such as fever, cough, chest pain, and fatigue. The infection often remains asymptomatic or resolves spontaneously within ten days. Radiographic findings in acute pulmonary histoplasmosis often include patchy pneumonitis and hilar lymphadenopathy [34]. Chronic pulmonary histoplasmosis can present in two forms: cavitary and non-cavitary. The cavitary form, previously thought to primarily affect male smokers with chronic obstructive pulmonary disease (COPD), is now known to occur in women and individuals without COPD as well. Cavities are typically located in the upper lobes, and sputum cultures often return positive results. Nodules, infiltrates, and mediastinal lymphadenopathy on imaging characterize the non-cavitary form. Although tissues contain the yeast, they can often contain the infection. Approximately 40% of chronic pulmonary histoplasmosis cases involve cavities [43]. Extrapulmonary dissemination can occur in acute and chronic infections, particularly in immunocompromised individuals. Disseminated histoplasmosis can affect various organs, including the mediastinum, skin, and central nervous system. This form of the disease is most common in individuals with severely weakened immune systems, such as those with advanced HIV infection [44]. The distinction between acute and chronic presentations is crucial for effective management. Acute infections are usually self-limited but can cause severe symptoms and dissemination in immunocompromised hosts. Chronic infections, particularly chronic cavitary histoplasmosis, require treatment to prevent progressive lung damage. Chronic cavitary disease can persist for months to years if untreated and may be mistaken for tuberculosis or other infections, underscoring the importance of accurate diagnosis and appropriate treatment [45].

Diagnosis

A comprehensive patient history is crucial for establishing a clinical suspicion of histoplasmosis. Key factors include geographic exposure to endemic regions like the Ohio and Mississippi River valleys. Occupation and recreational activities that involve contact with soil, exploration of caves, or exposure to environments with bird droppings significantly increase the risk of infection [39]. In addition, documenting respiratory symptoms such as cough, chest pain, and fever, as well as systemic symptoms like weight loss and night sweats, can guide the clinician toward a potential diagnosis. A thorough physical examination may reveal signs such as fever, lymphadenopathy, or respiratory distress, further supporting the suspicion of histoplasmosis [39]. Laboratory tests are essential for confirming the diagnosis of histoplasmosis. Serological tests for antibodies against *H. capsulatum *can be informative but may yield false negatives in acute cases and are less reliable in immunocompromised patients [46]. Antigen detection tests, which analyze urine and serum samples, have gained prominence due to their rapid results and high sensitivity, particularly for diagnosing acute and disseminated forms of the disease. Fungal culture remains the gold standard for diagnosis, involving the isolation of the organism from blood, sputum, or tissue samples, although this process can take several weeks. Histopathological examination of tissue samples, such as lung biopsies, can provide definitive evidence of infection by revealing the yeast form of *H. capsulatum *under the microscope [46]. Imaging studies are integral to the diagnostic process. A chest X-ray is typically the initial imaging modality used to assess pulmonary involvement, with findings that may include nodules, infiltrates, cavitary lesions, or hilar lymphadenopathy. A CT scan may be employed for more detailed visualization, offering a clearer picture of lung abnormalities and allowing for a more thorough assessment of disease extent, particularly in complicated or chronic cases [47]. Given that histoplasmosis can mimic other conditions, differential diagnosis is crucial. Tuberculosis is a primary concern due to similar respiratory symptoms and radiographic findings; distinguishing between the two often requires a thorough history and specific cultures [48]. Other fungal infections, such as coccidioidomycosis, blastomycosis, and cryptococcosis, may present overlapping symptoms. Bacterial pneumonia, especially community-acquired pneumonia, can have similar clinical presentations, making culture and antigen testing vital for differentiation. In addition, conditions like lymphoma or sarcoidosis, which can present with pulmonary symptoms and lymphadenopathy, require careful evaluation. Viral infections, such as influenza or COVID-19, may also present with respiratory symptoms, necessitating precise history-taking and testing for accurate diagnosis [48].

Management

Managing histoplasmosis requires a comprehensive approach that includes antifungal therapy, careful monitoring of treatment duration, and addressing any complications that may arise [49]. Antifungal therapy is prescribed based on the severity of the disease. Itraconazole is the first-line treatment for mild to moderate pulmonary histoplasmosis, including acute and chronic forms in immunocompetent individuals. It is also effective for disseminated histoplasmosis, which does not involve the central nervous system (CNS). In contrast, amphotericin B is reserved for severe or life-threatening cases, particularly in immunocompromised patients or those with significant organ involvement. It is often used initially for severe cases before transitioning to itraconazole for maintenance therapy [49]. The duration of antifungal treatment depends on the infection's severity. Itraconazole is typically administered for six to 12 weeks for mild cases, with the exact duration based on clinical response and symptom resolution. In severe cases, patients may receive amphotericin B for one to two weeks, followed by a switch to itraconazole for a total treatment duration of six to 12 months, especially in cases of disseminated histoplasmosis. Monitoring during treatment is crucial and includes regular clinical assessments to evaluate symptom resolution and potential side effects from antifungal therapy. Laboratory tests, such as liver function and serum drug levels, are also monitored, particularly for itraconazole, to ensure therapeutic levels and minimize toxicity [50]. Histoplasmosis can lead to several complications requiring specific management strategies. One such complication is fibrosing mediastinitis, which may require surgical intervention if it causes significant symptoms or complications, such as airway obstruction. In some cases, corticosteroids may be used to reduce inflammation associated with this condition. Another potential complication is pericarditis, which can be managed with anti-inflammatory medications, including non-steroidal anti-inflammatory drugs (NSAIDs) or corticosteroids. In severe cases, drainage of pericardial effusion may be necessary to alleviate symptoms and prevent further complications [51].

Prognosis and Outcomes

Histoplasmosis, even in immunocompetent individuals, involves various prognostic factors and outcomes crucial for effective management and follow-up. Understanding these factors helps clinicians provide optimal care and anticipate potential complications [45,52]. Several key prognostic factors significantly influence the outcomes of histoplasmosis in immunocompetent individuals. Age is a critical factor, with older patients generally exhibiting higher mortality rates associated with the disease. Studies indicate that the mean age of immunocompetent patients with histoplasmosis is often higher than that of their immunocompromised counterparts, correlating with increased severity and mortality. In addition, the presence of underlying health conditions, such as diabetes or chronic lung disease, can adversely affect prognosis. Immunocompetent individuals with these chronic conditions may experience worse outcomes compared to healthier patients. The severity of illness at presentation is another important prognostic factor; those presenting with severe respiratory distress or requiring intensive care are more likely to have higher mortality rates. Lastly, the initial response to antifungal therapy is crucial in determining long-term outcomes. Patients who respond well to treatment generally have a better prognosis [53]. Long-term follow-up is essential for patients recovering from histoplasmosis, as it enables healthcare providers to monitor recovery and identify potential long-term complications. Research indicates that most patients show significant improvement after several weeks of treatment, with mucosal healing and overall recovery rates improving over time [50]. While the prognosis is generally favorable, recurrence rates in immunocompetent individuals are relatively low, estimated at around 5%. Recurrences are more common in patients with underlying health issues or those immunocompromised during the initial infection. Factors such as incomplete antifungal therapy and the recovery of immune function post-treatment also influence the likelihood of recurrence [54].

Comparison of coccidioidomycosis and histoplasmosis

Coccidioidomycosis and histoplasmosis are endemic fungal infections that, while presenting with similar clinical features, have distinct differences in diagnosis, management, and regional considerations. Recognizing these similarities and differences is crucial for effective patient care and public health strategies [55]. Both infections primarily manifest as respiratory illnesses, often mimicking community-acquired pneumonia [56]. Common symptoms include fever, cough, chest pain, and fatigue. Both coccidioidomycosis and histoplasmosis can also lead to extrapulmonary manifestations, particularly in disseminated forms. Coccidioidomycosis may present with skin lesions, while histoplasmosis can affect the liver and spleen in severe cases. Risk factors for both infections overlap significantly, as environmental exposure, especially activities that disturb soil, such as construction or farming, increases the likelihood of inhaling fungal spores. In addition, residing in or traveling to endemic areas significantly heightens the risk of infection for both diseases [56].

Despite these similarities, coccidioidomycosis and histoplasmosis differ notably in their diagnostic approaches and management strategies. Diagnosis of coccidioidomycosis often involves serologic tests for antibodies or antigen detection, with imaging studies sometimes revealing solitary nodules that may be confused with malignancies, necessitating further investigation [57]. By contrast, histoplasmosis diagnosis typically relies on antigen detection in urine or serum, which is both rapid and sensitive. Chest imaging for histoplasmosis often shows multiple small, calcified nodules, which can help differentiate it from coccidioidomycosis. Management strategies also differ: mild cases of coccidioidomycosis may require only observation, while moderate to severe cases are treated with fluconazole or itraconazole. Severe cases may initially require amphotericin B, followed by a transition to oral antifungals. For histoplasmosis, moderate to severe cases typically begin with amphotericin B due to the organism's high sensitivity, with step-down therapy often involving itraconazole for less severe cases [58]. Regional considerations are pivotal in the differential diagnosis and management of these infections. Coccidioidomycosis is primarily found in the southwestern United States, particularly in arid regions like Arizona and California. This geographic specificity necessitates a high index of suspicion in these areas when patients present with respiratory symptoms. By contrast, histoplasmosis has a broader geographic distribution in the central and eastern United States, particularly in areas with high nitrogen soil content [58]. This wider prevalence means that clinicians must consider histoplasmosis in a broader range of patients, especially those with a history of exposure to endemic areas. Public health strategies must adapt accordingly: in areas endemic to coccidioidomycosis, increasing clinician education and awareness is essential for improving timely diagnosis and management. Surveillance and reporting systems are critical for monitoring disease trends and outbreaks. For histoplasmosis, public health initiatives often focus on educating at-risk populations about exposure risks and seeking medical attention for respiratory symptoms [59]. A comparison of coccidioidomycosis and histoplasmosis in immunocompetent individuals is shown in Table 1.

**Table 1:** Comparison of coccidioidomycosis and histoplasmosis in immunocompetent individuals

Feature	Coccidioidomycosis	Histoplasmosis
Causative agent	Caused by the dimorphic fungi* Coccidioides immitis *and *Coccidioides posadasii*, which exist in soil as mold.	Caused by the dimorphic fungus *Histoplasma capsulatum*, commonly found in soil enriched with bird or bat droppings.
Geographical distribution	Primarily endemic to arid regions such as the Southwestern United States (Arizona, California) and parts of Central and South America.	Commonly found in the Ohio and Mississippi River valleys in the United States, with cases also reported in parts of Central and South America, Africa, and Asia.
Mode of transmission	Inhalation of airborne arthroconidia (spores) released from disturbed soil, particularly after events like dust storms or earthquakes.	Inhalation of microconidia from soil contaminated with bird or bat droppings, often during activities like caving, construction, or farming.
Incubation period	The incubation period ranges from one to three weeks after exposure, with symptom onset varying based on the individual's immune status and extent of exposure.	The incubation period typically ranges from three to 17 days after exposure, with symptoms manifesting sooner in cases of high exposure.
Clinical presentation	It often starts with flu-like symptoms, including cough, fever, fatigue, and chest pain. Some individuals may develop erythema nodosum or erythema multiforme. In more severe cases, it can progress to chronic pulmonary coccidioidomycosis with cavitary lesions or disseminated disease affecting the skin, bones, joints, and meninges.	Presents with similar respiratory symptoms such as fever, cough, and chest pain. Acute histoplasmosis can resemble flu or pneumonia, while chronic histoplasmosis resembles tuberculosis with symptoms like weight loss and night sweats. Disseminated histoplasmosis is more common in immunocompromised individuals but can occur in immunocompetent persons, affecting organs such as the liver, spleen, and central nervous system.
Radiographic findings	Chest X-rays or CT scans may show nodules, cavities, or diffuse infiltrates, particularly in the upper lobes. In disseminated cases, bone lesions and soft tissue abscesses can also be seen.	Imaging may reveal hilar or mediastinal lymphadenopathy, nodules, or cavitary lesions, particularly in the lungs. Calcified granulomas or mediastinal fibrosis may be seen in chronic cases.
Diagnosis	Diagnosis is often confirmed through serological testing for Coccidioides antibodies (IgM and IgG), direct microscopy, culture of respiratory or tissue samples, and PCR for fungal DNA. Skin testing with coccidioidin or spherulin is less commonly used today.	Diagnosis is based on serology (detection of antibodies), antigen testing (urine, blood), culture of respiratory or tissue samples, and histopathology showing yeast forms inside macrophages. PCR can also be used for rapid detection.
Treatment	Mild cases may resolve without treatment, but antifungal therapy is recommended to prevent complications. First-line treatment includes fluconazole or itraconazole for mild to moderate cases, while severe or disseminated infections may require amphotericin B followed by oral azoles for maintenance.	Most cases are self-limiting, but antifungal therapy is indicated for more severe or chronic forms. Itraconazole is the drug of choice for mild to moderate disease, with amphotericin B used for severe or disseminated infections. Chronic cases may require long-term antifungal therapy.
Prognosis in immunocompetent individuals	The prognosis is generally favorable in immunocompetent individuals, with most cases resolving completely. However, some may experience relapses or develop chronic forms of the disease if left untreated. Dissemination, though rare, can occur and requires aggressive treatment.	The prognosis is good for most immunocompetent individuals, with many cases resolving without treatment. However, chronic or disseminated histoplasmosis, while less common in this group, can occur and requires prompt antifungal therapy to avoid serious complications.
Prevention	Preventive measures include avoiding activities that disturb soil in endemic areas, wearing masks during high-risk activities, and using dust control methods in construction or agricultural settings. Public health education is important in endemic regions.	Prevention strategies involve avoiding exposure to environments with high concentrations of bird or bat droppings, especially in endemic areas. Wearing protective equipment during activities like caving or demolition work can reduce the risk of infection. Public awareness and environmental control measures are key in high-risk regions.

Future directions and research gaps

Advancements in diagnostic techniques are crucial for enhancing the identification and management of coccidioidomycosis and histoplasmosis. Emerging molecular diagnostics, such as polymerase chain reaction (PCR) assays, are showing promise in detecting specific DNA sequences of *Coccidioides *and *Histoplasma *species, offering superior sensitivity and specificity compared to traditional methods [60]. In addition, identifying fungal biomarkers, including galactomannan and (1,3)-β-D-glucan, holds the potential for improving diagnosis, especially in immunocompromised individuals. The role of point-of-care testing is also gaining traction, with rapid and accurate testing facilitating timely treatment initiation. The development of lateral flow assays for antigen detection has shown potential, although further validation is needed to assess their effectiveness across various clinical settings [61]. Regarding treatment, novel therapeutic approaches are being explored to enhance patient outcomes. New antifungal agents, including novel azoles and echinocandins, are under development and may offer improved efficacy and safety profiles compared to existing therapies. Immunotherapy and adjunctive treatments are also being investigated to boost the host immune response, particularly in severe or refractory cases. Therapies targeting specific cytokines or immune pathways, such as interferon-gamma and interleukin-12, may present new treatment avenues, especially for patients with underlying immunodeficiencies [61]. Epidemiological studies are vital for understanding the dynamics of coccidioidomycosis and histoplasmosis. Continuous surveillance in both endemic and non-endemic regions is essential for monitoring incidence, prevalence, and geographic distribution. This data guides public health interventions and informs clinicians about the likelihood of these diseases in specific areas. Moreover, the impact of climate change on the incidence and spread of these fungal infections is an emerging area of concern. Research is needed to explore how environmental factors, such as temperature, humidity, and soil composition, influence the ecology and transmission of *Coccidioides *and *Histoplasma *species [55]. Public health implications are significant, making effective prevention and education strategies essential for reducing the burden of coccidioidomycosis and histoplasmosis. Increasing awareness among healthcare providers about clinical presentations, diagnostic methods, and management strategies is crucial. Public education campaigns targeting high-risk populations, such as outdoor workers and travelers to endemic areas, can help prevent exposure and promote early diagnosis [62]. Addressing challenges in resource-limited settings is imperative, as these infections often disproportionately affect populations with limited access to healthcare and diagnostic resources. Developing cost-effective and sustainable diagnosis and treatment strategies and strengthening healthcare infrastructure will be key priorities in these environments [63]. Future directions and research gaps in coccidioidomycosis and histoplasmosis are summarized in Table 2.

**Table 2:** Future directions and research gaps in coccidioidomycosis and histoplasmosis

Area of focus	Coccidioidomycosis	Histoplasmosis
Epidemiology	- Need for updated and comprehensive epidemiological data across different regions, including non-endemic areas. - Exploration of climate change impacts on the geographical spread of *Coccidioides *species.	- Comprehensive studies to better understand the epidemiology in non-endemic regions. - Investigation into the impact of changing environmental conditions on the prevalence of *Histoplasma capsulatum*.
Diagnosis	- Development of rapid, point-of-care diagnostic tests with high sensitivity and specificity. - Research into more accessible and cost-effective diagnostic tools for resource-limited settings.	- Improvement in rapid diagnostic methods, particularly in low-resource settings. - Enhanced accuracy of antigen detection assays and development of non-invasive diagnostic tools.
Treatment	- Exploration of novel antifungal agents with fewer side effects and better efficacy against resistant strains. - Research into personalized treatment approaches based on genetic and immunological factors.	- Development of new antifungal therapies targeting resistant or refractory cases. - Studies on optimizing treatment regimens to reduce treatment duration and side effects.
Vaccine development	- Ongoing research to develop a safe and effective vaccine for Coccidioidomycosis. - Investigation into the immunological mechanisms required for protective immunity.	- Research focused on developing vaccines, particularly for high-risk populations. - Understanding the immune response in histoplasmosis to inform vaccine development.
Public health interventions	- Implementation and evaluation of community-based education programs in endemic areas. - Research into the effectiveness of preventive measures in high-risk occupations and activities.	- Developing public health strategies to reduce exposure risk, particularly in endemic regions. - Assessment of the impact of public awareness campaigns on reducing incidence rates.
Pathogenesis	- Deeper understanding of the pathogenesis at the molecular level, including host-pathogen interactions. - Research into the mechanisms of immune evasion by Coccidioides species.	- Further studies on the molecular pathogenesis and host immune response. - Investigation into the factors leading to severe and disseminated histoplasmosis in immunocompetent individuals.
Genomic studies	- Genomic studies to identify virulence factors and resistance genes in Coccidioides species. - Research into the genetic diversity of the pathogen in various geographical regions.	- Genomic analysis to identify virulence factors and resistance mechanisms in Histoplasma capsulatum. - Studies on genetic variations among different strains and their clinical implications.
Clinical trials	- More randomized controlled trials (RCTs) to compare different treatment modalities and durations. - Research into the long-term outcomes of treated versus untreated individuals.	- Need for large-scale RCTs to evaluate the efficacy of existing and new treatments. - Studies on long-term outcomes and management of chronic and relapsing histoplasmosis.
Dissemination and chronic disease	- Investigation into the predictors of disease dissemination and chronicity in immunocompetent individuals. - Research into long-term management strategies for chronic coccidioidomycosis.	- Understanding the factors that contribute to chronic and disseminated disease in immunocompetent individuals. - Development of management guidelines for chronic histoplasmosis.

## Conclusions

Coccidioidomycosis and histoplasmosis, although often associated with immunocompromised individuals, pose significant health risks to immunocompetent populations as well. These fungal infections, endemic to specific geographic regions, can lead to a wide range of clinical manifestations, from mild respiratory symptoms to severe, life-threatening conditions. The rising incidence of these infections in non-endemic areas highlights the importance of increased awareness and improved diagnostic capabilities. A thorough understanding of the clinical features, diagnostic challenges, and management strategies is essential for ensuring timely and effective treatment. As research continues to advance, it is crucial to develop more refined therapeutic approaches and public health strategies to address the evolving landscape of these infections. By doing so, we can enhance patient outcomes and better manage the public health impact of coccidioidomycosis and histoplasmosis in immunocompetent individuals.
